# Analyzing gene expression profiles in dilated cardiomyopathy via bioinformatics methods

**DOI:** 10.1590/1414-431X20164897

**Published:** 2016-10-10

**Authors:** Liming Wang, L. Zhu, R. Luan, L. Wang, J. Fu, X. Wang, L. Sui

**Affiliations:** 1 Emergency Department, The Second Affiliated Hospital of Xi'an, Jiaotong University, Xi'an, China; 2 Department of Emergency Medicine, The First Affiliated Hospital of Harbin Medical University, Harbin, China; 3 Medical Department, The First Affiliated Hospital of Harbin Medical University, Harbin, China

**Keywords:** Dilated cardiomyopathy, Differentially expressed genes, Pathway enrichment analysis, Transcription factors, microRNAs, Small molecules

## Abstract

Dilated cardiomyopathy (DCM) is characterized by ventricular dilatation, and it is a common cause of heart failure and cardiac transplantation. This study aimed to explore potential DCM-related genes and their underlying regulatory mechanism using methods of bioinformatics. The gene expression profiles of GSE3586 were downloaded from Gene Expression Omnibus database, including 15 normal samples and 13 DCM samples. The differentially expressed genes (DEGs) were identified between normal and DCM samples using Limma package in R language. Pathway enrichment analysis of DEGs was then performed. Meanwhile, the potential transcription factors (TFs) and microRNAs (miRNAs) of these DEGs were predicted based on their binding sequences. In addition, DEGs were mapped to the cMap database to find the potential small molecule drugs. A total of 4777 genes were identified as DEGs by comparing gene expression profiles between DCM and control samples. DEGs were significantly enriched in 26 pathways, such as lymphocyte TarBase pathway and androgen receptor signaling pathway. Furthermore, potential TFs (SP1, LEF1, and NFAT) were identified, as well as potential miRNAs (miR-9, miR-200 family, and miR-30 family). Additionally, small molecules like isoflupredone and trihexyphenidyl were found to be potential therapeutic drugs for DCM. The identified DEGs (PRSS12 and FOXG1), potential TFs, as well as potential miRNAs, might be involved in DCM.

## Introduction

Dilated cardiomyopathy (DCM) is characterized by the dilatation of the myocardium, generally associated with systolic dysfunction. DCM, which affects the left ventricle more often than the right, commonly leads to heart failure, and it can result in arrhythmias, thromboembolism and premature death (1). The etiology of DCM is multifactorial, and involves idiopathic, familial, genetic, viral or immune factors (2,3). Nowadays, the molecular mechanism of DCM is still unclear due to its heterogeneity. Therefore, exploring the underlying mechanism of DCM and searching for potential genes involved in DCM are of great significance to human health.

Previous studies revealed that genes, transcription factors (TFs) and microRNAs (miRNAs) are involved in DCM. Mutation in some genes, such as *TNNT2*, has been suggested to cause DCM (4). The dysregulation of gene expression, like in prospero-related homeobox factor 1 (*PROX1*), is also involved in DCM. *PROX1* can directly repress the expression of fast-twitch skeletal muscle genes (e.g., troponin T3, troponin I2, and myosin light chain 1) at transcriptional level (5). Cardiac-specific knockout of *PROX1* causes overexpression of fast-twitch genes, and thus leads to a change from slow-twitch to fast-twitch muscle phenotype, as well as postnatal development of fatal DCM (5). Moreover, *GATA4* is one of the cardiac TFs crucial for normal cardiogenesis, and its heterozygous mutations, like p.V39L, p.P226Q and p.T279S, have been found in sporadic DCM patients (6). These mutants can decrease the transcription regulatory activity of *GATA4* and reduce the synergistic activation between *NKX2-5* and *GATA4* (6). In addition, miRNAs like miR-1, miR-29c, miR-30c, miR-30d, miR-149, miR-486, miR-499 are down-regulated in murine phospholamban mutant model of DCM, and the individual silencing of these miRNAs can contribute to cardiac cell loss and heart failure (7).

As a powerful technique, gene expression microarray analysis based on bioinformatics has been widely applied to identify DCM-related genes, possible molecular functions, and biological signal pathways. Based on independent microarray datasets like GSE3585, GSE3586, and GSE1869, a gene expression signature consisting of 27 genes (e.g., *MYH6*, *MYH10*, *CCL2*, *PHLDA1*, *SNCA*, *FRZB*, *SFRP4*, *SPOCK*, *CTGF*, *G0S2*, *ETV5*, and *RARRES1*) has been identified for DCM, as well as the down-regulation of immune response processes (8). By integrating gene expression profiles with protein-protein interaction (PPI) network analysis, Lin et al. (9) have constructed specific co-expressed PPI networks for DCM and non-DCM samples. Hub proteins in the DCM network tend to be differentially expressed, and two DCM-related functional modules (muscle contraction and organ morphogenesis) have also been identified (9). Using the microarrays of GSE3586, Xiao et al. (10) identified the functional modules related to heart failure with different etiologies. However, these studies focused mainly on genes, and little is said about the TFs or miRNAs that regulate gene expression, or about the potential molecular drugs for DCM treatment.

In this study, we re-analyzed the gene expression profile of GSE3586 to explore the molecular mechanism of DCM at both the gene expression level and expression regulation level. Differentially expressed genes (DEGs) were identified between DCM and normal samples. DEGs bio-functions, potential TFs, and potential miRNAs, as well as small molecules that could be employed in the treatment of DCM, were also investigated.

## Material and Methods

### Microarray data

The gene expression profile of GSE3586 (8) was obtained from the Gene Expression Omnibus (GEO) database (http://www.ncbi.nlm.nih.gov/geo/) based on the platform of Human Unigene 3.1 cDNA Array 37.5K v1.0. A total of 28 samples were available, including 13 DCM samples from septal myocardial tissue of DCM patients and 15 normal samples from non-failing donor hearts of healthy controls.

### Data preprocessing and DEGs screening

As the intrinsic background of different chips might affect the calculation of expression values, the raw data of each chip were first normalized using the Geoquery package (version 2.34.0, available at http://www.bioconductor.org/packages/release/bioc/html/GEOquery.html) (11) in R language. After, the expression data were log2 transformed, and a linear regression model was constructed to compare the gene expression in DCM and control samples. Then, the Benjamini and Hochberg (BH) procedure (12) was applied to adjust P values and thus obtain false discovery rate (FDR). The Limma package (13) in R language was used to identify the DEGs in DCM and control samples. Finally, the log2 fold change (FC) ≠0 and FDR <0.05 were chosen as the cutoff values.

### Hierarchical clustering analysis

For the identified DEGs, hierarchical clustering analysis was performed by using the pheatmap package (version 1.0.2, available at http://cran.r-project.org/web/packages/pheatmap/index.html) created by R Core Team (Austria).

### Pathway analysis of DEGs

All the metabolic and non-metabolic pathways that involved DEGs were analyzed by using the Gene Set Analysis Toolkit V2 (WebGestalt2) platform (Vanderbilt University, Nashville, TN, USA; available at http://bioinfo.vanderbilt.edu/webgestalt) (14) based on the Wikipathways database (15). P values less than 0.05 and the number of involved genes ≥2 were selected as cut-off criteria.

### Potential TFs and miRNAs

TFs and miRNAs play crucial roles in DCM. The Molecular Signature Database version 3.0 (MSigDB 3.0, available at http://www.broadinstitute.org/msigdb) (16) contains hallmark, positional, curated, motif, and computational gene sets, gene ontology, oncogenic signatures, and immunologic signatures. Among these, motif gene sets include genes that share a conserved and cataloged cis-regulatory motif in promoters and in 3′-untranslated regions (3′-UTRs). Specifically, motif gene sets contain TF targets that share a TF binding site recorded in the TRANSFAC database (version 7.4, http://www.gene-regulation.com/), as well as miRNA targets that share a 3′-UTR miRNA binding motif. In this study, based on the well-annotated motif gene sets in MSigDB 3.0, Gene Set Enrichment analysis (GSEA) (17) was performed to identify the potential TFs and miRNAs of DEGs by utilizing hypergeometric distribution. The criterion was set as FDR <0.05.

### Identification of potential small molecules associated with DCM

Currently, the connectivity map (cMap) database includes 7056 gene-expression datasets that involve 6100 small molecule treatment-control pairs (18). In order to identify the small molecules that could simulate the normal or DCM cells, the identified DEGs (up- and down-regulated genes) were mapped to the small molecules deposited in cMap database using GSEA (17). The correlation score was calculated, ranging from -1 to 1.

## Results

### Screening, hierarchical clustering, and pathway analysis of DEGs

**Figure 1:**
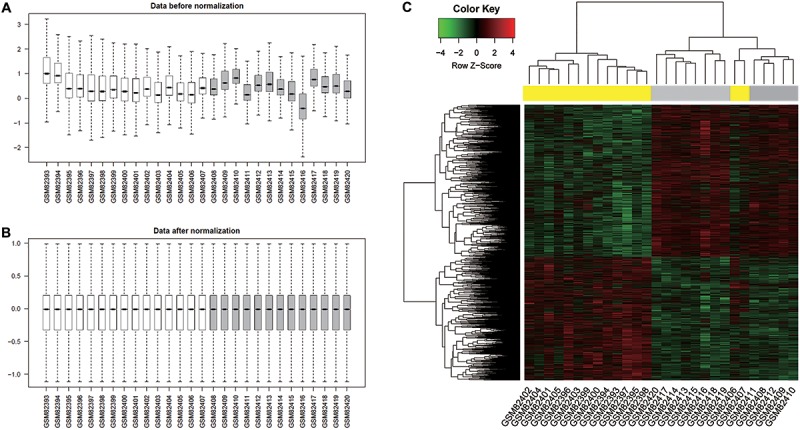
Normalization of gene expression data and hierarchical clustering of differentially expressed genes (DEGs). *A*, gene expression profiles before normalization. White boxes represent normal samples, while gray boxes stand for dilated cardiomyopathy samples. *B*, gene expression profiles after normalization. *C*, hierarchical clustering of DEGs. Yellow boxes represent normal samples, while gray boxes stand for dilated cardiomyopathy samples. The green and red bars represent low and high expression levels, respectively. GSMxxxxx: the accession number of a certain sample in the Gene Expression Omnibus database.

After data normalization (Figure 1A and B) and differential expression analysis, a total of 4777 DEGs were identified in DCM and normal samples, including 2711 up-regulated genes and 2066 down-regulated genes. Genes like *PRSS12* (protease serine 12, log2 FC=-0.167 and FDR=0.003) and *FOXG1* (forkhead box G1, log2 FC =-0.156 and FDR=0.045) were significantly down-regulated in DCM samples in comparison with normal samples. In addition, the expressions of 13 DEGs were elevated more than 2-fold, while only 1 gene (*CCL2*) was down-regulated more than 2-fold. For the identified DEGs, hierarchical clustering analysis was performed, and samples were classified into two clusters (Figure 1C). Surprisingly, 2 normal samples were clustered with DCM samples rather than with normal samples. This might be caused by intrinsic deviation of the clustering tool (namely, the pheatmap package), potential sub-healthy state of control individuals, and experimental deviation. In general, samples in normal and DCM groups could be distinguished based on DEGs expression. After pathway analysis based on Wikipathways database (15,19), a total of 26 significant pathways were identified (Table 1), among which 8 pathways were associated with signaling and 2 pathways were involved in cancer. A total of 124 DEGs (e.g., *POLA2*, *TMED7*, *SLC25A24*, *NF2*, and *COL4A2*) were significantly enriched in "lymphocyte TarBase pathway" (P value=9.35×10^-16^), while 28 DEGs (e.g., *NCOR2*, *DSTN*, *RAC1*, *PIK3R1*, and *FHL2*) were significantly enriched in "androgen receptor signaling pathway" (P value= 9.12×10^-7^).

**Table:**
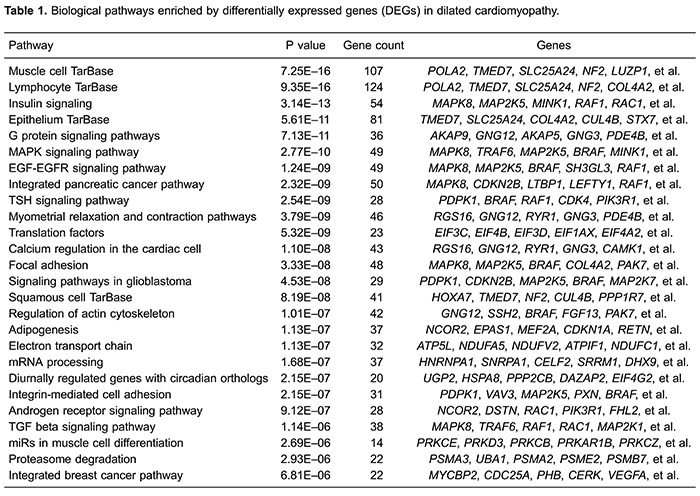

### Potential TFs and miRNAs

The top 10 target sites and corresponding TFs with a highly significant correlation are listed in Table 2, and *SP1*, lymphocyte enhancer factor-1 (*LEF1*), and nuclear factor of activated T cells (NFAT) were among the most significant TFs. *SP1* and *LEF1* collectively targeted 191 genes; *LEF1* and NFAT collectively targeted 149 genes, like down-regulated *PRSS12*; *SP1* and *NFAT* collectively targeted 147 genes; *SP1*, *LEF1*, and NFAT collectively targeted 58 genes.

**Table:**
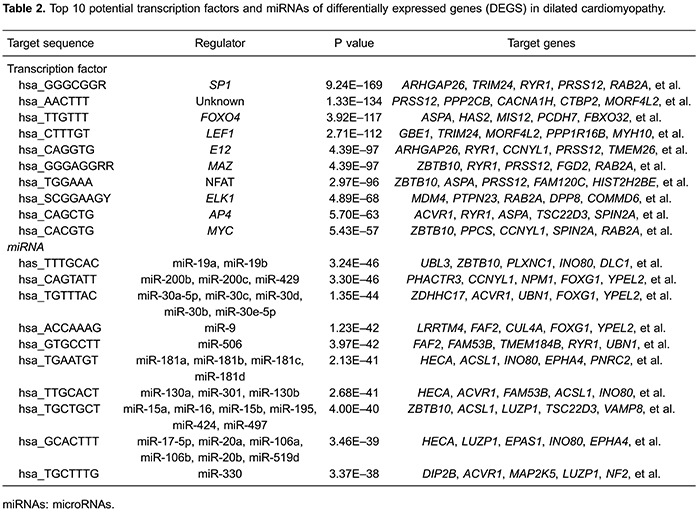

In addition, the potential miRNAs of DEGs were screened, and miR-9, miR-200 family, and miR-30 family were among the most significant miRNAs (Table 2). More specifically, miR-9, miR-200 family, and miR-30 family collectively targeted the down-regulated *FOXG1*.

### Potential small molecules associated with DCM

Based on the cMap database, we performed GSEA for DEGs to screen for possible small molecule drugs. Finally, 20 small molecules were identified as having a highly significant correlation with DCM (Table 3), including 6 negatively correlated and 14 positively correlated small molecules. Among these molecules, isoflupredone and trihexyphenidyl could be potential small molecule drugs for DCM treatment, and DL-thiorphan and milrinone might trigger DCM (Table 3).

**Table:**
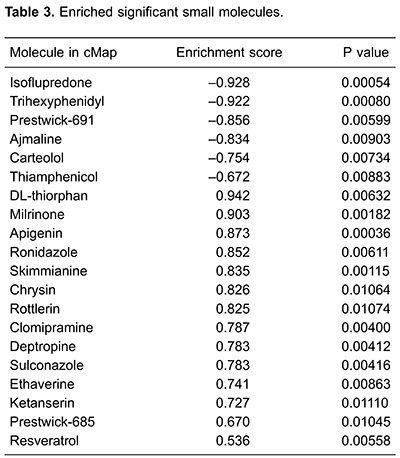

## Discussion

DCM is characterized by ventricular dilatation, and it commonly leads to heart failure. Although many studies have been devoted to exploring the pathogenesis of DCM, the mechanism of DCM development and progression still remains largely unknown. In the present study, we identified the DEGs between DCM and normal samples, performed pathway analysis for DEGs, and predicted potential TFs, miRNAs, and small molecules related with DCM.

Bioinformatics gene expression microarray analysis has been widely used to identify DCM-related genes. Based on the microarrays of GSE3586, Barth et al. (8) found 1353 up-regulated transcripts and 384 down-regulated transcripts by using 2-class unpaired significance analysis of microarrays with the criteria of FDR <0.05 and fold change ≥1.2. However, we screened out 4777 DEGs by utilizing Limma package with the criteria of FDR <0.05 and log2 FC≠0. The differences in DEGs number between our study and the previous study might be caused by the different analysis methods and criteria; however, our method and criteria are the most commonly used in DEG screening.

Moreover, the previous study has proven that the immune response process is involved in end-stage DCM (8), and we identified that the lymphocyte TarBase pathway and androgen receptor signaling pathway were significantly enriched by DEGs between DCM and normal samples. Reportedly, the lymphocyte is an important part of the immune system and has a vital role in heart function (20). Neutrophil/lymphocyte ratio is associated with the low function capacity in DCM patients (21). In androgen receptor signaling pathway, the androgen receptor is involved in the inflammation response and affects myocardial function directly (22). Thus, we suggested that lymphocyte TarBase pathway and androgen receptor signaling pathway might play roles in DCM via immune system.

Generally, TFs can play pivotal roles in regulating the transcription process of encoding genes. In this study, we also predicted potential TFs of DEGs, such as *LEF1*, NFAT, and *SP1*. Being expressed in pre-B and T lymphocytes, *LEF1* is a regulatory participant in lymphocyte gene expression and differentiation (23). The transcriptional activity of *LEF1* is related to heart function via plakoglobin (24). Besides, the translocation of β-catenin-TCF/*LEF-1* complex into the nucleus is involved in Wnt/wingless signal transduction pathway activated by ATP depletion to modulate the expression of genes, which can regulate cell proliferation, apoptosis, and differentiation (25,26). Thus, we speculated that *LEF1* might be related with DCM. In addition, NFAT is expressed in immune-system cells and plays a vital role in the transcription of cytokine genes and other genes which were critical for the immune response (27). NFAT is also a critical regulator of cardiac development and myocyte maturation (28), and the translocation of dephosphorylated *NFAT-3* to nucleus in DCM can activate the calcineurin signaling pathway (29), whose activation can induce atrial hypertrophy during atrial fibrillation (30). Specifically, *LEF1* and NFAT collectively targeted the down-regulated PRSS12, which is also named neurotrypsin or motopsin. Reportedly, neurotrypsin can produce C-terminal agrin fragment that plays crucial roles in the initiation and maintenance of neuromuscular junctions and is a biomarker of muscle wasting in congestive heart failure patients (31). This evidence indicated that these TFs might play roles in DCM. Also, *SP1* can regulate the expression of NF-κB related cytokines, and is associated with inflammation in aortic aneurysm and heart failure (32). Also, *SP1* participates in androgen receptor signaling pathway. Although there is very few evidence showing that *SP1* is involved in DCM, we speculated that *SP1* might associate with DCM, as *SP1* and NFAT collectively targeted 147 genes.

miRNAs can post-transcriptionally regulate gene expression, and act as pivotal modulators in DCM (7). In this research, potential miRNAs of DEGs were identified, and miR-9 was the most significant one. miR-9 can regulate NFATc3-mediated hypertrophy signaling (33), and NFAT was identified in our study. Gladka et al. (34) reported that miR-9 was involved in dilated cardiac hypertrophy on a regulatory perspective, and miRNAs in miR-200 family are related with DCM and heart failure (35). miR-200a can regulate β-catenin expression and subsequently modulate Wnt/β-catenin signaling (36), while there is little information that focuses on the function of miR-200b or miR-200c in DCM. Besides, miR-30c and miR-30d are down-regulated in murine model of DCM, and their silencing contributes to cardiac cell loss and heart failure (7). Specifically, miR-9, miR-200 family, and miR-30 family collectively targeted the down-regulated *FOXG1*, which belongs to the forkhead box family, and the deletion of Foxm1 leads to diminished DNA replication and mitosis in cardiomyocytes, and ventricular hypoplasia in Foxm1^-/-^ mouse line (37). Therefore, we speculate that miR-9, miR-200 family, and miR-30 family might play vital roles in DCM.

Furthermore, the expression patterns of DEGs between normal and DCM samples were mapped to cMap database, and a series of small molecules were predicted to correlate with DCM. Small molecules isoflupredone and trihexyphenidyl were found to be potential drugs for the prevention and treatment of DCM. Isoflupredone is mainly used in inflammatory diseases, and it is effective in the endotoxin-induced mastitis and can significantly improve lung function in inflammatory airway disease (38,39). Additionally, trihexyphenidyl is considered a treatment option for idiopathic cervical dystonia (40), while it is unknown whether trihexyphenidyl has effects on DCM. Therefore, it needs to be further researched whether these two small molecules are useful in treating DCM.

In conclusion, our study identified DEGs between DCM and control samples (e.g., *PRSS12* and *FOXG1*), and these DEGs participated in significant pathways such as lymphocyte TarBase pathway and androgen receptor signaling pathway. Potential TFs *(LEF1*, *SP1* and NFAT) and miRNAs (miR-9, miR-200 family, and miR-30 family) might play roles in DCM. Furthermore, two small molecules (isoflupredone and trihexyphenidyl) might be capable of treating DCM. This result might provide new insight into understanding the molecular mechanism of DCM and finding new therapeutic targets of DCM.

More experiments are needed to verify these results, as they were obtained through bioinformatics analysis. We are planning to perform gene overexpression and silencing analysis to investigate the roles of potential DCM-related genes, transcription factors, and miRNAs in DCM. Moreover, we are also planning to use animal models to study the therapeutic effects of the potential drugs isoflupredone and trihexyphenidyl, identified in this research.
